# Umbilical Hemorrhage

**Published:** 1896-08

**Authors:** H. B. Granberry

**Affiliations:** Austin, Texas


For the Texas Medical Journal.

### UMBILICAL HEMORRHAGE.

BY H. B. GRANBERRY, M. D., AUSTIN, TEXAS.

[Read at Austin District Medical Society, June 21, 1896.]

IN LITERATURE this subject is almost completely ignored, and in practice but little spoken of—not that it is not worthy of thought, or that it is not a source of thought as deep and real as any that can present itself in adult life. The protection of infants, and the expenditure of medical as well as surgical means to secure that protection, are subjects that justly invite the research and expression of experience of any medical fraternity. To accomplish the latter is the sole object and purpose of this paper. In many cases, it can be said, the causes of umbilical hemorrhage, "like the stone of Sisyphus, rolls back from whence it came," and are largely the fruits of the indiscretions and sins of man.

To be convinced that there is truth in this, it is only necessary to separate the indiopathic or spontaneous from the traumatic or accidental hemorrhage. The one coming from the capillaries or oozing tissues of the naval stump,—with no apparent cause; the other from the umbilical vessels themselves, and a direct result of a too careless tying of the suture about the cord, or an undue pulling and jerking of the cord, due to roughly handling the child and the pulling of an improperly applied abdominal bandage, which at times proves a source of self-reproach and the making of a sad home.

When there is a persistent disposition to bleed from the capillaries, with an obstinate oozing from the tissues of the navel itself, accompanied by a bleeding from the umbilical vessels, it is far more serious, and is indicative of, or rather should cause a suspicion of the existence of a grave condition—that of hæmophelia morbus maculosus or a hemorrhagic diathesis. These terms are synonymous, and represent a vicious constitutional derangement, in which there is such profound impressions made upon the blood and blood vessels that the one refuses to deposit a firm fibrinous coagula to occlude the vessels, while the other, meaning the umbilical vein, hypogastric and umbilical arteries have walls so wanting in contractibility that they remain patulous, and thereby permit hemorrhage to occur.

There is a cause for every effect, yet to search for and endeavor to explain with any degree of certainty a cause in the fœtus itself a utero, must necessarily be, if not "far fetched and foolish," unsatisfactory, at least, as our means of ascertaining is poor, and not without fallacy—however, I am inclined to think that this disease, for it is a disease, can not occur only from an unhealthy lineage.

Acquired causes, those that come on after delivery and originate in the child itself, irrespective of maternal or paternal impressions, can be credited only with question. There is usually such a short interval from birth to the appearance of hemorrhage that it is not irrational to conclude that there are but few instances which have no hereditary taint. A child beginning life with a comparative degree of health can not undergo such a marked change in so short a time (with scarcely an exception) as to have its nervous system so completely dethroned, its blood made so entirely wanting in coagulability, and its vessels in contractility as to produce a fatal hemorrhage—with a cause solely originating in itself, and that, too, after delivery.

Perhaps an exception that can be cited is that of hepatic origin, it being a recognized fact that bleeding is a symptom of jaundice, and that jaundice is a cause of bleeding. It is of but little moment whether it is due to a hepatitis, a condition, as some claim, can follow an inflammation produced by the ligature about the cord, a congenital occlusion of the bile duct, or from any cause, altering the blood, which in turn affects the vessels and tissues to so great an extent as to invite this condition; as Silberman claims, by impoverishing the blood by a fibrin ferment, which interferes with the forming of a firm coagulum or clot. The red blood corpuscles, under the microscope of Pònfric and Silberman, show a disintegration, thus a clot with such a frame-work of fibrin, and a body of disintegrated blood corpuscles can only be recognized as fragile and dangerous to life.

The hereditary causes of umbilical hemorrhage are of far more importance, as but few, as has been previously stated, occur that have no ancestral history that predisposes. The taints of the mother are more likely to be transmitted than those of the father, for her blood is the store-house from which the child receives, during gestation, its nutrition. Her poor health, therefore, protracted by any disease, increases the fluidity of her blood, and robs it of its nutritive principle, which necessarily places the child in a low medium of life, insuring it flabby vessels and impoverished blood. Excesses in alcoholic stimulants on the part of the mother not only destroys the vital force in the red blood corpuscles, but induces a dilutation of the peripheral blood vessels, a condition transmitted to the child without variance, and bringing about a non-contractible disposition of the vessel walls. The most potent factor in bringing about a hemorrhagic diathesis is, beyond queston, syphilis. For frequency it is sadly great, and its effect certain in breaking down the high nutritive principle of the blood, and as Sir Simpson claims, it sets up an artritis, followed by thickening of vessels, causing them to lose their tonicity. It is, then, necessary to imagine or wonder at the vaso-constrictors failing to respond, for there can be no response in the elastic or contractile coats of the vessels under a condition of such low vitality; it is contrary to nature, yet we deal with nature.

Hemorrhage may be subcutaneous, from mucous surfaces or the umbilical stump, yet all such hemorrhages are not syphilitic, notwithstanding congenital syphilis passes through all the usual stages of syphilis save the primary sore. When the hemorrhage is subcutaneous it is due, beyond question, to an interference of local circulation by morbid products, which causes a sanguinous effusion and purpuric spots. When of a mucous or umbilical origin (as Behrings claims), it is due to diminished coagulability of the blood, and to a vascular stasis, especially of the small cutaneous vessels. Whether it can occur in utero is a question that can not be answered with any degree of certainty, for no literature or tabulated experience of any one shows that the cord, blackened with contused blood, extending some distance from the umbilicus, or the purpuric spots found on the body of the newly born, are due other than to traumatism during delivery. Knowing, however, the anatomical condition of the child immediately before and shortly after birth are markedly the same, it is not a strained conclusion to believe that a per cent. of mortality in utero, with this radically changed condition of the cord at birth, is due to this diathesis. Purpura and idiopathic bleeding are sypmtomatic rather than disease; consequently, when purpuric spots are seen on the body of the child, with ancestrial history of bleeders or syphilis, we may suspect that bleeding will come on. However, with no degree of certainty can this suspicion be cherished. Why not come on immediately after delivery? Stephen Smith, with a report of 79 cases, and Foster Jenkins with 178, shows that the largest per cent. takes place between the fifth and seventh day. It is true that at this time most cords "drop off," but this fact bears no direct relationship to the purpuric spots, yet these spots on the other hand indicate the presence of hæmophelia, and are produced by a morbid product in the blood. Why does it occur even before the line of separation is established between the ligated cord and the naval stump? for cases are reported as taking place on the day after delivery. And why is it that it likewise occurs days after the loss of the cord? These questions can all be answered by considering the loss of coagulability of the blood and want of tonicity of the vessels.

Why not come at once after delivery? Because the body of the child, formerly protected by the lubricating influence and even temperature of the embryonic fluid when born—is thrust into a new medium as it were—the constantly varying temperature of the atmosphere, and the friction from clothing, which produces an effect upon the endings of the censory nerves—which in turn acts by reflex upon the vaso-constrictors, thus preventing hemorrhage. It is plausable that, after a short time, varying in individual cases, the contractile muscles of the arterial walls—worn out by the effect of contraction, finally refuse to respond to the sensation of the vaso-constrictors (and like an arm held in prolonged extension, drops to the side in sheer exhaustion) thus permitting a dilation of the vessels—the blood, then, an impoverished heritage, wanting in coagulability, fails to come to the rescue with a thrombus, and leaves the little one an easy prey to fate.

The mortality is great, yet death does not usually occur at the first bleedings. The anæmia soon following, necessarily disturbs the digestive system, making convalescence very slow. The smaller the amount and the longer the intervals between bleedings, the more rapidly it does, and the more favorable it is for the child to recover.

Treatment involves not only a consideration of the immediate danger of the acute stage of hemorrhage, but that of prevention, to avoid, if possible an occurrence of the bleeding—meaning, treat the child in utero by treating the parental diathesis, whether it be some latent cause of a strumous origin, indicating for the mother tonics, exercise and fresh air—or of a specific nature with syphilis as a background, requiring anti-syphilitic treatment. Parents with a history of bleeding children should be treated. Hæmaphelite is not really detected until bleeding begins, for at birth there may be no signs to indicate such—so treatment usually begins when hemorrhage begins.

The cord pulsation ceases first at the placenta, consequently it should not be cut too near the body from a standpoint of guarding against any hemorrhage that might be liable to occur.

Rest and position are of importance—the one with its quieting influence over the heart, the other (on back) enabling the lungs to expand with the greatest amount of freedom, which is one of nature's hæmostatics, by way of lessening blood pressure in the descending aorta and hypogastric arteries.

The salines are effective in reducing general blood pressure; of these sodium phosphate is perhaps preferable, on account of its saline properties and more complete action in reducing the portal circulation, by influencing the biliary secretions. Pressure by a sponge or tampon saturated with stiptic drugs, held firmly about the body of the child by a plain, or an Esmarch bandage carefully applied, are the usual resorts. The lattter too tightly used provokes vomiting and endangers the lower extremities by interfering with circulation; therefore it is well to bear in mind that gentle pressure accomplishes all that can be accomplished by more violent means, and reduces to a minimum the liability of bringing about a gangrenous state of the lower extremities. Plaster paris moulds held firmly to the child has its advocates, as does the various stiptics in combination with collodion.

Ergot and tincture per chloride of iron internally are of value in strumous cases, and some of the mercurials in the syphilitic, the most acceptable and effective being the ointment of the red iodide. Fowler's solution of arsenic pushed to a physiological effect is of marked value in convalescence, due to its effect on the vascular walls.

Pins, passed at right angles through the stump with a figure of eight ligature thrown about the pins is a common surgical resort. Better than this is the passage of a large thread subcutaneously about the stump and firmly tied, obliterating completely the umbillical vessels.

To more fully accomplish the purposes of this paper, in conclusion I will cite a case which has proved of much interest to me and impressed me with the importance of treatment in utero.

A Mrs. A., age 21 or 22, of German descent, on January 1st, 1895, gave birth to a still-born girl baby. She was in the hands of a mid-wife. Found head of child in lower straight, resting on the perinum. Delivery was soon accomplished, the cord presenting for at least a foot from the body of the child a congested and almost black condition. Placenta was removed with no difficulty at all, and convalescence took place rapidly. On December 9th, same year, the lady was confined again. Labor was easy and rapid, and a well developed girl baby was born. Mother and child did well until the beginning of the third day, when I was called, to find the little one with umbilical hemorrhage. A tampon saturated with tannic acid was applied and held in position by abdominal bandage. Tincture per chloride of iron was afterward used on tampon with ergot given internally, also a saline. Afterwards a styptic collodion dressing was used—and getting no beneficial result, acetanelede was tried; then a ligature thrown entirely about the stump—realizing the fact that sloughing of the stump might have been produced. This failing, subcutaneous ligature was resorted to, which controlled the hemorrhage, only to be followed in a few hours by bleeding from the mouth and nose, resulting in death. Investigation showed the mother with a history of good health, extending to her grand parents. The father was syphilitic and a bleeder.

We should investigate not only parental history, but the history of children of previous deliveries, and direct our treatment accordingly.

